# Genetic dissection of the relationships between grain yield components by genome-wide association mapping in a collection of tetraploid wheats

**DOI:** 10.1371/journal.pone.0190162

**Published:** 2018-01-11

**Authors:** Giacomo Mangini, Agata Gadaleta, Pasqualina Colasuonno, Ilaria Marcotuli, Antonio M. Signorile, Rosanna Simeone, Pasquale De Vita, Anna M. Mastrangelo, Giovanni Laidò, Nicola Pecchioni, Antonio Blanco

**Affiliations:** 1 Department of Soil, Plant & Food Sciences, Genetics and Plant Breeding Section, University Aldo Moro, Bari, Italy; 2 Department of Agricultural & Environmental Science, Research Unit of “Genetics and Plant Biotechnology”, University Aldo Moro, Bari, Italy; 3 Council for Agricultural Research and Economics—Cereal Research Centre, Foggia, Italy; Institute of Genetics and Developmental Biology Chinese Academy of Sciences, CHINA

## Abstract

Increasing grain yield potential in wheat has been a major target of most breeding programs. Genetic advance has been frequently hindered by negative correlations among yield components that have been often observed in segregant populations and germplasm collections. A tetraploid wheat collection was evaluated in seven environments and genotyped with a 90K SNP assay to identify major and stable quantitative trait loci (QTL) for grain yield per spike (GYS), kernel number per spike (KNS) and thousand-kernel weight (TKW), and to analyse the genetic relationships between the yield components at QTL level. The genome-wide association analysis detected eight, eleven and ten QTL for KNS, TKW and GYS, respectively, significant in at least three environments or two environments and the mean across environments. Most of the QTL for TKW and KNS were found located in different marker intervals, indicating that they are genetically controlled independently by each other. Out of eight KNS QTL, three were associated to significant increases of GYS, while the increased grain number of five additional QTL was completely or partially compensated by decreases in grain weight, thus producing no or reduced effects on GYS. Similarly, four consistent and five suggestive TKW QTL resulted in visible increase of GYS, while seven additional QTL were associated to reduced effects in grain number and no effects on GYS. Our results showed that QTL analysis for detecting TKW or KNS alleles useful for improving grain yield potential should consider the pleiotropic effects of the QTL or the association to other QTLs.

## Introduction

Increasing grain yield in cereal crops has been a major goal of most breeding programs, and the effects of genetic improvement on yield potential has been reported in several studies [1–6]. Improving grain yield has always been a difficult task as it is a typical quantitative trait controlled by several genes, strongly influenced by environmental factors and crop management. In the last five decades, genetic gains in common and durum wheat have been mainly obtained by increasing harvest index associated to the gradual reduction in plant height. Further improvement are expected by increasing the biomass production and the radiation use efficiency without any reduction of the harvest index of modern cultivars [7–10]. Anyway, grain yield potential is the final product of plant growth and development, and several others complex factors, such as abiotic stress tolerance, adaptation to different soils and climate changes, disease resistances, contribute to plant productivity.

The primary numerical components of grain yield are the number of spikes per unit area, the average number of kernel per spike (KNS) and the average kernel weight, usually determined as one-thousand kernel weight (TKW). The product of kernel number and kernel weight is grain yield per spike (GYS). The importance of TKW also derives from being a marketing standard directly related to milling quality. KNS and TKW are quantitatively inherited, while the number of spikes per unit area, in the intensive cropping systems, mainly depends from planting density. Several authors (e.g. [11–12]) reported that an increase in seeding densities is almost invariably associated with a linear increase of the number of spikes per square meter across a wide range of seed rates.

Genetic advance in wheat breeding has been frequently hindered by negative correlations among yield components that have been often observed in segregating populations and germplasm collections (see reviews [6,13]). Particularly, the phenotypic correlation between KNS and TKW has been generally found negative even though not always consistent [14–16], while the correlations between GYS and the sub-components KNS and TKW have been always found consistent and positives. Phenotypic correlations may be attributed to genetic linkage, pleiotropy, environmental factors, and yield component compensations due to competition for a common limited nutrient supply. One of the most likely and widely accepted hypothesis for explaining the negative relationship between grain number and grain weight is that the increase of grain number per unit area or per spike produced by a genotype results in a lower availability of photo-assimilates synthesized during grain filling for each grain, which leads to decreases in individual grain weight due to competition effects [13,17–18]. Numerous results in the literature (e.g. [7,19–21]) on the effects of the introduction of the *rht* genes in breeding lines and in the released semi-dwarf cultivars during the 1960s and 1970s demonstrated that grain yield progress was associated with the increased number of grains per square meter and the increased number of kernels per spike. The same studies found a tendency of a concomitant decrease of grain weight in the widely cultivated semi-dwarf varieties. However, some investigations on common wheat [22–23] underlined how in most conditions the availability of assimilates was not the limiting factor for grain growth, and the negative relationship between KNS and TKW could not be attributed to competition among grains for limited assimilates. Moreover, recently studies [6,24–27] reported that in different countries grain yield progress of advanced breeding lines and cultivars was mainly associated with increased grain weight.

The recent development and increased availability of high throughput genotyping technologies have significantly contributed to the genetic dissection of complex traits into discrete quantitative trait loci (QTL). The classical QTL mapping approach is conducted in segregating populations resulting from crossing two parental lines different for the trait of interest, and exploiting the genetic association between molecular markers and QTL [28]. However, the development of large biparental populations is time-consuming, QTL analysis can allow detecting loci in genomic regions containing polymorphisms restricted to the parental lines, and the resolution power is rather low due to the limited number of crossing-over and recombination. The recent, alternative approach of the genome-wide association study (GWAS), based on the linkage disequilibrium present in natural germplasm (landraces, breeding lines and elite cultivars), provides higher resolution in QTL analysis and increased power in loci with modest-size effects detection [29–30]. The limitation of GWAS in detecting a high frequency of false-positive and false-negative marker-trait associations is usually overcome by appropriate statistical methodologies, which take into account the population structure and relative kinship among individuals, and the multiple testing of thousands markers [31].

Mapping studies for the several components of grain yield potential have identified QTL on all 21 chromosomes of wheat genome by classical linkage mapping using biparental populations and by GWAS using wheat collections (see reviews [32–34]). Major QTL associated to thousand grain weight were detected on chromosomes 1B, 2A, 2D, 3A, 3B, 4B, 4D, 5A, 5B, 6A, 7B and 7D [16,35–52], and QTL for grain number per spike on chromosomes 2B, 3B, 4A, 4B, 4D, 5A, 5D, 7A and 7B [33,36–38, 43,48,53–59]. Current development of advanced biotechnologies have recently allowed the identification of some key genes for significant increases in grain weight or grain number per spike in wheat by comparative genomics with rice [60].

A better understanding of the genetic architecture of yield components is particularly necessary to break the negative relationship between number of KNS and TKW, and then to achieve further genetic progress in wheat breeding programs. The objectives of this study were: a) to identify major and stable QTL for KNS and for TKW by GWAS in a tetraploid wheat collection evaluated in several environments; b) to analyse the genetic relationships between GYS, KNS and TKW at QTL level; c) to identify molecular markers tightly linked to grain yield components. The identification of stable QTL that increase one component without decreasing others across environments is essential for improving wheat grain yield by traditional and genomic selection programs.

## Materials and methods

### Plant materials and phenotypic trait evaluation

The tetraploid wheat (*Triticum turgidum* L., 2n = 4x = 28; AABB genome) collection used in this study was comprised of 233 accessions (S1 Table) chosen to represent the phenotypic variability for the grain yield component traits that were evaluated in this study. The panel, including wild and cultivated accessions of seven subspecies (*durum*, *turanicum*, *polonicum*, *turgidum*, *carthlicum*, *dicoccum* and *dicoccoides*), has been characterized in terms of genetic diversity and population structure [61] and used for genome-wide association mapping of loci controlling agronomic traits [62] and some qualitative traits, such as β-glucan content [63] and carotenoid content [64].

The whole wheat collection was grown in southern Italy in the experimental fields of the University of Bari at Valenzano (Bari) for five years (2009, 2010, 2012, 2013 and 2014, hereafter reported as V09, V10, V12, V13, V14), at Foggia in 2012 (hereafter reported as F12) and at Gaudiano (Potenza) in 2013 (hereafter reported as G13). A randomized complete block design with four replications (V09, V10) or three replications (V12, F12, V13, G13, V14) and plots consisting of 1-m rows, 30 cm apart, with 50 germinating seeds per plot, was used in all field experiments. During the growing season, 100 kg/ha of N was applied and standard cultivation practices were adopted. Plots were hand-harvested at maturity and GYS was determined dividing grain yield per row by the number of spikes per row (about 70–80 spikes). A 15-g seed sample per plot was used to determine the TKW. KNS was calculated as the ratio between the average GYS and the average TKW.

### DNA extraction and SNP genotyping

Genomic DNA was isolated from freeze-dried young leaf tissues using the protocol as described by Sharp et al. [65]. DNA quality was checked by electrophoresis on 1.0% agarose gels, and the concentration determined with a NanoDrop spectrophotometer. DNA of each sample was diluted to 50 ng μl^-1^ and genotyped for single-nucleotide polymorphism (SNP) using the wheat 90K Infinium iSelect array containing 81,587 gene-associated SNP markers [66]. Genotyping procedure was performed at TraitGenetics Laboratory, Gatersleben, Germany (http://www.traitgenetics.de) following the manufacturer’s recommendations as described in Akhunov et al. [67]. The genotyping assays were carried out to the Illumina iScan reader and performed using Genome Studio software version 2011.1.

### Statistical and association mapping analyses

In the current study, each year-location combination was considered as an environment. Standard procedures for analysis of variance for each yield component was carried out with MSTAT-C software. Genetic variance (*σ*^*2*^_*G*_), environmental variance (*σ*^*2*^_*E*_), and variance due to genotypic x environment interaction (*σ*^*2*^_*G x E*_) were obtained by using the combined analysis of variance. Broad-sense heritability (*h*^*2*^_*B*_) was estimated by the ratio *σ*^*2*^_*G*_*/σ*^*2*^_*P*,_ where *σ*^*2*^_*P*_ is phenotypic variance (*σ*^*2*^_*P*_ *= σ*^*2*^_*G*_ *+ σ*^*2*^_*G x E*_ *+ σ*^*2*^_*E*_). Pearson phenotypic correlation coefficients were calculated between GYS, TKW and KNS.

SNP markers with >10% missing data points and markers with a minimum allele frequency (MAF) of less than 5% were removed from the data matrix prior to GWAS. Association between SNP markers and individual grain yield component traits was tested by using: a) the general linear model (GLM) and the GLM including the Q-matrix derived from the principal component analysis (PCA) as implemented in TASSEL (GLM+Q), and b) the mixed linear model (MLM) based on the kinship-matrix (MLM+K) and the MLM based on both the K-matrix and the Q-matrix (MLM+K+Q). Marker-trait associations (MTA) were considered significant at threshold–log_10_(P)≥3.0 determined by the modified Bonferroni correction as implemented in Genstat software [68]. In agreement to the linkage disequilibrium (LD) estimates determined by Laidò et al. [62], the value of 8 cM was used as the support interval to declare significant SNPs associated with the examined yield component traits. QTL for GYS, KNS and TKW were considered stables when detected at–log_10_(P)≥3.0 in at least three environments or two environments and the mean across environments, while were considered suggestive QTL at the sub-threshold 2.0<–log_10_(P)<3.0.

The consensus high-density linkage map of durum wheat described by Maccaferri et al. [69] was used as reference map for chromosome localization and map position of SNP markers associated to QTL for each trait. The proportion of phenotypic variance explained by a single QTL was determined by the square of the partial correlation coefficient (*R*^*2*^%). Additive effect for the bi-allelic SNP markers was estimated by TASSEL as the difference of the phenotypic effect between the most frequent allele toward the less frequent allele; positive or negative sign indicates the increasing or decreasing effect of the SNP allele with higher frequency. Graphical representation of linkage groups and QTL was carried out using MapChart 2.2 software [70].

## Results

### Phenotypic variation of grain yield components

**Fig 1:**
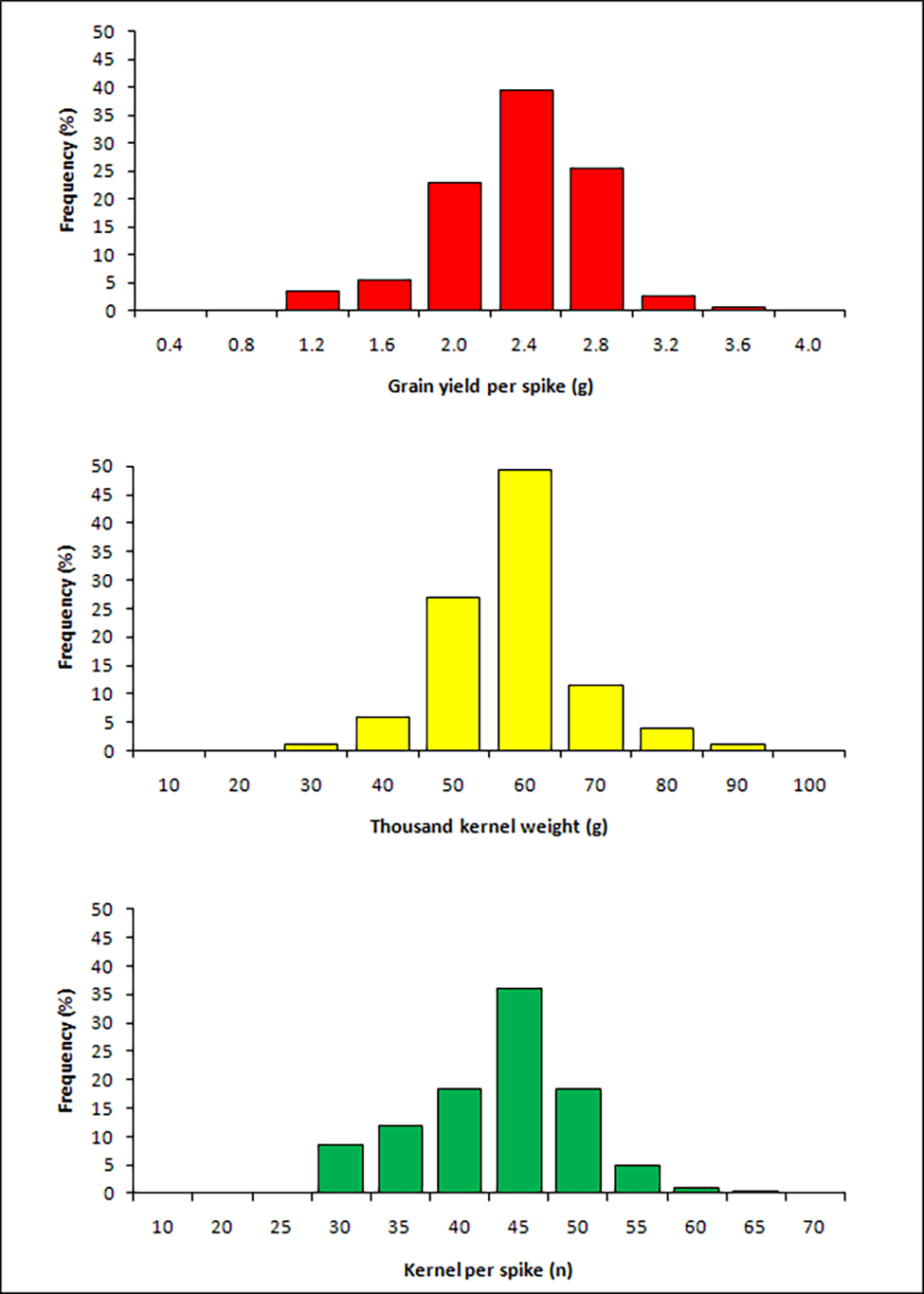
Frequency distributions of mean values of grain yield per spike (g), thousand-kernel weight (g) and kernel number per spike (n) in a tetraploid wheat collection evaluated in seven field experiments.

Analysis of variance revealed highly significant differences between genotypes for GYS, KNS and TKW in each environment, while the combined analysis across environments revealed significant effects of genotypes, environments, and a strong genotype x environment interaction (G by E) (S2 Table). Mean GYS ranged from 1.76 g to 2.52 g in each environment with the average GYS across the seven environments being 2.08 g (Table 1). Mean TKW ranged from 46.7 to 59.5 g and mean KNS from 35.1 to 46.2 in each environment. The wide range of each trait in each environment can be attributed to the composition of the collection, which include wild and semi-domesticated accessions, breeding lines and modern cultivars. The normal distribution pattern and the large variation indicated the polygenic control of the examined yield components in the tetraploid wheat collection (Fig 1). Estimates of broad sense heritability showed high values in individual environments, but relatively low values for GYS (0.52) and KNS (0.49) and high for TKW (0.78) across environments.

**Table 1:** Means, ranges, coefficients of variation (CV), genetic variance (*σ2G*) and heritability (*h2B*) of grain yield per spike, kernel number per spike and thousand-kernel weight in a tetraploid wheat collection evaluated in seven environments.

Traits	Environments							Across environments
	V09	V10	F12	V12	V13	G13	V14	
*Grain yield per spike (g)*								
Mean	1.95	2.19	2.52	1.76	2.30	2.12	2.01	2.08
Range	(0.44–3.24)	(0.70–3.59)	(0.70–4.28)	(0.52–3.08)	(0.74–3.62)	(0.74–3.48)	(0.81–3.38)	(0.80–3.25)
CV	15.9	11.3	13.2	14.7	8.8	9.0	11.2	11.3
*σ*^*2*^_*G*_	0.207	0.305	0.391	0.235	0.287	0.269	0.238	0.164
*h*^*2*^_*B*_	0.68	0.83	0.78	0.78	0.88	0.88	0.82	0.52
*Kernel per spike (n)*								
Mean	41.7	41.3	46.2	36.0	41.9	35.1	42.6	40.3
Range	(13.5–63.7)	(21.8–61.3)	(20.5–88.9)	(16.8–58.1)	(23.8–82.0)	(18.1–63.3)	(20.8–66.5)	(20.7–62.5)
CV	17.9	8.7	12.2	12.9	8.2	8.8	9.5	9.6
σ^2^_G_	55.862	55.795	102.625	63.228	64.360	48.215	66.188	39.554
h^2^_B_	0.70	0.81	0.91	0.75	0.85	0.84	0.80	0.49
*Thousand-kernel weight (g)*								
Mean	46.7	52.8	54.9	48.8	54.3	59.5	46.9	51.3
Range	(24.3–73.5)	(26.5–88.8)	(26.4–86.2)	(26.3–74.9)	(25.3–88.3)	(29.5–93.5)	(21.4–78.9)	(41.2–61.1)
CV	16.9	5.5	5.4	5.4	4.5	4.5	5.9	4.9
σ^2^_G_	80.789	105.128	104.848	73.451	123.981	151.705	105.630	81.698
h^2^_B_	0.92	0.93	0.92	0.91	0.95	0.95	0.93	0.78

Phenotypic correlations between the three yield components in each environment are reported in Table 2. GYS was significantly positively correlated with KNS (*r* values ranging from 0.61 to 0.83) and TKW (*r* from 0.53 to 0.71) in all environments and across environments. Correlations between KNS and TKW achieved at each environment ranged from statistically not significant values (*r* = 0.06 to -0.11) to negative and statistically significant coefficients (*r* = -0.23 at V14). KNS was negatively related to TKW across environments at 0.01P (*r* = -0.22).

**Table 2:** Correlations between grain yield per spike (GYS), thousand-kernel weight (TKW) and kernel number per spike (KNS) in a tetraploid wheat collection evaluated in seven environments.

Environment	GYS-TKW	GYS-KNS	TKW-KNS
V09	0.63***	0.70***	-0.02
V10	0.713***	0.72***	0.06
F12	0.53***	0.83***	0.00
V12	0.59***	0.70***	-0.12
V13	0.66***	0.66***	-0.11
G13	0.68***	0.69***	-0.05
V14	0.57***	0.65***	-0.23**
Across environments	0.63***	0.61**	-0.22**

**, ***: Significant at P<0.01 and P<0.001, respectively

### Detection of QTL for grain yield components

**Fig 2:**
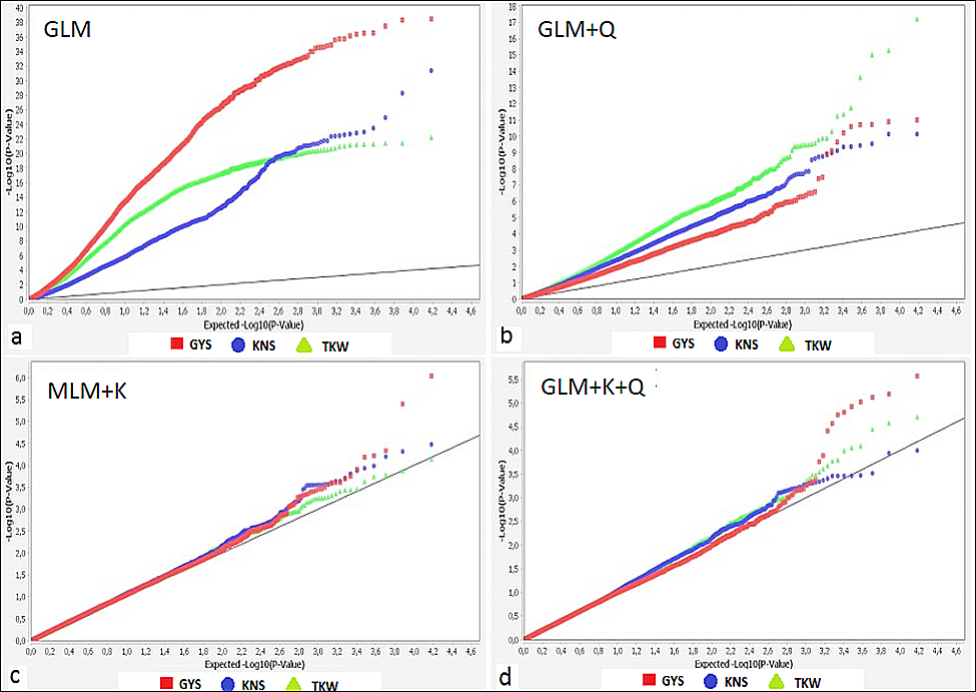
Quantile-quantile plots by four statistical GWAS models for yield component traits (mean values across seven environments): a) general linear model (GLM); b) GLM including the Q-matrix derived from the principal component analysis (GLM+Q); c) mixed linear model (MLM) based on the kinship-matrix (MLM+K); d) MLM based on both the K-matrix and the Q-matrix (MLM+K+Q). GYS = Grain yield per spike, KNS = Kernel number per spike, TKW = Thousand kernel weight.

The tetraploid wheat collection was analysed with the 90K iSelect genotyping array including 81,587 gene-associated SNPs recently developed by Wang et al. [66]. A total of 15,211 markers mapped in the durum consensus map [69] were retained for the GWAS analysis after removing failed and monomorphic markers, SNPs with more than 10% of missing data and SNPs with a minor allele frequency lower than 0.05. Marker-trait associations were determined by four statistical models: the GLM, the GLM+Q model taking into account the population structure (determined by the principal component analysis), and the MLM incorporating the K matrix (MLM+K) and the K and Q matrices (MLM+K+Q) in order to consider the confounding effects of both population structure and relative kinship, and minimize type 1 errors (false-positive associations). Inspection of Q-Q plots and Manhattan plots for each yield component trait in each environment and across environments (Fig 2 and S1 Fig) indicated strong deviations of the observed -log_10_(P) values from the expected -log_10_(P) distributions for the GLM and GLM+Q models, while the closer observed and expected distributions of the -log_10_(P) values in the MLM+K and MLM+K+Q models suggested the reduction of potential spurious marker-trait associations. The last two models produced similar results and the MLM+K model was definitively used in the GWAS analysis.

**Fig 3:**
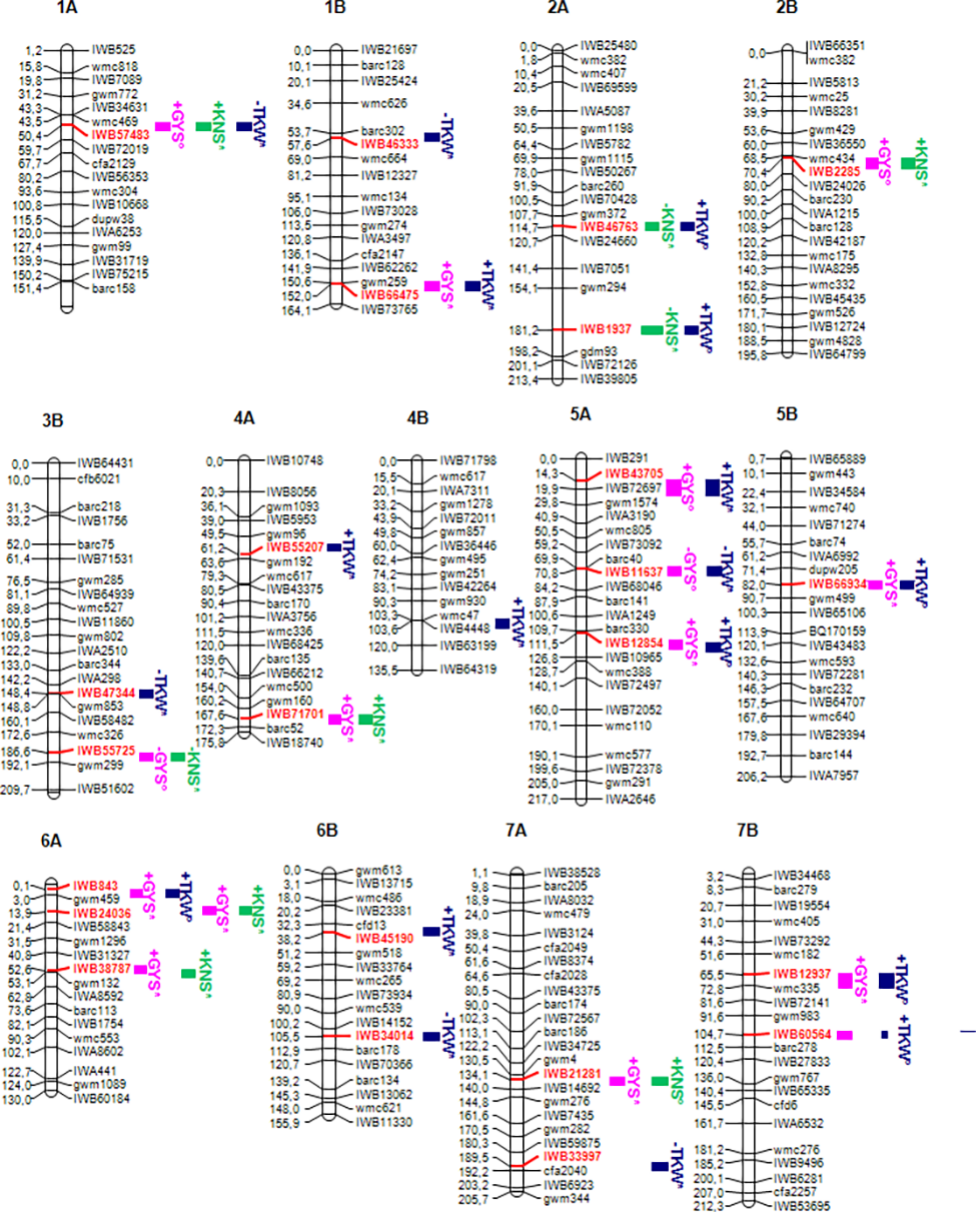
Schematic representation of A and B genome chromosomes of the durum consensus linkage map [69] with map positions of QTL for grain yield component traits. Each chromosome map is represented by the first and the last SNP marker, and by a SNP marker every about 20 cM. SSR markers have been also inserted every about 20 cM to compare the consensus SNP map with published SSR-based maps. Markers are indicated on the right side and cM distances on the left side of the bar. QTLs are represented by bars on the right of each chromosome bar. QTL names indicate the trait (GYS for Grain Yield per Spike, TKW for Thousand Kernel Weight, KNS for Kernel Number per Spike). The closest SNP marker is indicated in red.

Several QTL significant at–log_10_(P)≥3.0 in one or two environments were detected for each grain yield component on all 14 chromosomes; these QTL were considered environment-specifics and not reported in the present work because we were interested in stable QTL potentially useful for wheat breeding programs. QTL for GYS, KNS and TKW detected at–log_10_(P)≥3.0 in at least three environments or two environments and the mean across environments are reported in S3 Table and illustrated in Fig 3. As one of the objective of the present work was to analyze the variation of one component in relation with the other traits change, *P* value, additive effect and *R*^*2*^ value for the three yield components were reported for each significant stable QTL across all environments. Ten stable QTL were detected for GYS, individually accounting for 5.0 to 9.4% of the phenotypic variation and consistent at -log_10_(P) ranging from 3.0 to 5.4. The QTL *QGys*.*mgb-5B* on the long arm of chromosome 5B was consistent in five environments and the mean across environments. Eight QTL were detected for KNS and 11 QTL for TKW. The–log_10_(P) scores for the KNS QTL ranged between 3.1 to 5.9, with each QTL explaining 5.0–11.6% of the phenotypic variation. The QTL for TKW were detected at–log_10_(P) from 3.0 to 5.6, each accounting 4.8–10.7% of phenotypic variation. All detected QTL were generally consistent at–log_10_(P)≥3.0 in 3–4 environments and in additional 1–3 environments at sub-threshold 2.0<–log_10_(P)<3.0, with additive effects in the same direction.

More interesting was the fact that among the eight QTL for KNS, six QTL (*QKns*.*mgb-1A*, *QKns*.*mgb-2B*, *QKns*.*mgb-3B*, *QKns*.*mgb-4A*, *QKns*.*mgb-6A*.*1*, *QKns*.*mgb-6A*.*2*) co-located with QTL for GYS at–log_10_(P)≥3.0 on chromosomes 4A and 6A (2 QTL), and at 2.0<–log_10_(P)<3.0 on chromosomes 1A, 2B and 3B. One additional suggestive KNS QTL was co-located with a significant GYS QTL on chromosome 7A. Among the 11 QTL for TKW, four QTL (*QTkw*.*mgb-1A*, *QTkw*.*mgb-1B*.*2*, *QTkw*.*mgb-5A*.*1* and *QTkw*.*mgb-5A*.*2*) co-located with QTL for GYS at–log_10_(P)≥3.0 on chromosomes 1A, and at 2.0<–log_10_(P)<3.0 on chromosomes 1B and 5A (two QTL). Five additional suggestive TKW QTL (*QTkw*.*mgb-5A*.*3*, *QTkw*.*mgb-5B*, *QTkw*.*mgb-6A*, *QTkw*.*mgb-7B*.*1* and *QTkw*.*mgb-7B*.*2*) were detected in the same chromosome location with significant QTL for GYS on chromosomes 5A, 5B, 6A and 7B (two QTL). Only the GYS QTL *QGys*.*mgb-1A* mapped with one QTL for both NKS and TKW (*QKns*.*mgb-1A* and *QTkw*.*mgb-1A*, respectively) on chromosome 1A (S3 Table).

## Discussion

During the last decades, grain yield improvement of wheat has been the major focus of most breeding programs in several countries of the world. Significant yield increases were achieved during the 1960s and 1970s transferring the semi-dwarfing *Rht* genes associated to reduced plant height, resistance to lodging, higher number of grain per spike e per unit area, and higher harvest index [71]. Later, the genetic progress obtained by selecting for yield *per se* has been weak, inducing hard effort for breeding work due to the quantitative nature of the trait controlled by a complex genetic system and the strong environmental factors and agronomic management influence. Grain yield increases using indirect selection of yield components has been difficult to achieve due to the complex interactions between plant developmental traits and yield components. Molecular markers are useful tools both for the genetic dissecting of complex traits and for analyzing the relationships of the different yield components at QTL level. Understand the genetic and physiological bases of grain yield may contribute to overcome the negative relationships among some components, and to develop appropriate and efficient breeding strategies for further yield improvement.

### Detection of stable QTL for yield components

The present study was designed to detect stable QTL for KNS and TKW in order to analyze the genetic basis of the interdependence between the above grain yield per spike sub-components in a tetraploid wheat collection evaluated in seven replicated field trials. The observed wide phenotypic variation for each yield component (Table 1) can be attributed to the composition of the collection including wild and semi-domesticated accessions, landraces and modern durum cultivars. A total of 15,211 SNP-derived genes were used for the genome wide-association study by using the GLM and the MLM models taking into account the confounding effect of population structure and the relative kinship. Q-Q plots (Fig 2) for the three examined yield-related traits indicated that the models MLM+K e MLM+K+PCs including the kinship matrix detected less potential spurious marker-trait associations than the models GLM and GLM+Q. Moreover, the MLM+K model performed slightly better than MLM+K+Q and was used as the most suitable model for the association mapping analysis of KNS, TKW and GYS, thus confirming other results of GWAS on grain quality traits carried out on the same tetraploid collection [64], and supporting previous findings on the efficiency of the MLM+K model for correcting cryptic relatedness for most of the traits [34,72].

Eighteen stable QTL for the two GYS sub-component traits, distributed on 13 of the 14 chromosomes of the A and B genomes, were detected at–log_10_(P)≥3.0 in at least three environments or two environments and the mean across environments (Fig 3 and S2 Table). Ten consistent QTL for GYS co-located with four KNS QTL and six TKW QTL. Each detected QTL was often consistent at sub-threshold 2.0>–log_10_(P)>3.0 in the remaining environments, and all had additive effects in the same direction. The consensus high-density linkage map for durum wheat described by Maccaferri et al. [69] was used in the current study as reference map for chromosome localization and map position of SNP markers associated to QTL. This consensus map includes both SNP and SSR markers and enabled us to compare the genomic regions involved in the quantitative expression of grain yield components found in the tetraploid wheat collection with map position of QTL found in previous analysis. Several experiments mapped QTL for yield components on all wheat chromosomes (see reviews by Araus et al. [32], Cui et al. [33] and Gupta et al. [34]. Major QTL associated to thousand grain weight were detected on chromosomes 1B, 2A, 2D, 3A, 3B, 4B, 4D, 5A, 5B, 6A, 7B and 7D [16,35–52], and major QTL for grain number per spike on chromosomes 2B, 3B, 4A, 4B, 4D, 5A, 5D, 7A and 7B [33,36–38,43,48,53–59]. Most QTL were detected in individual environments and/or single mapping population and will hardly be employed in wheat breeding programs with success. Differences in number and map position of QTL detected in the above studies may be attributed to the high number of effective genes controlling grain yield, the different genotypes of the mapping population parental lines, the G by E, the marker coverage of linkage maps used in QTL analyses, the statistical methodologies and the threshold values employed for the statistical significance of marker-trait associations.

While many of the QTL identified in the current study have been described previously, two stable QTL for KNS detected on chromosome arms 2AL and 3BL *(QKns*.*mgb-2A*, *QKns*.*mgb-3B*), and four stable QTL for TKW detected on 3BL, 6AS, 6BL and 7BL *(QTkw*.*mgb-3B*, *QTkw*.*mgb-6A*.*1*, *QTkw*.*mgb-6B*.*1*, *QTkw*.*mgb-7B*.*2)* were totally new. Moreover six QTL for KNS detected on chromosome arms 1AS, 2AL, 2BL, 4AL, 6AS, 6AL and 7AL, and eleven QTL for TKW detected on 1BS, 1BL, 4AL, 4BL, 5AS (three), 5BL, 6BS, 7AL and 7BS validated previously detected QTL in different genetic backgrounds, and they can be considered stable and useful for MAS in breeding programs. Interestingly, the QTL *QKns*.*mgb-4A* was repeatedly detected in several experiments in different environments and according to Cui et al. [58] should be subjected to map-based cloning.

Recently, some candidate genes for significant increases in grain weight were identified in wheat by comparative genomics with rice, such as *TaCwi-A1* [73]. *TaGS-D1* [74], *TaSus1* and *TaSus2* [75], *TaGS5-3A* [76], *TaCYP78-A3* [77], *TaCWI-5D* [78], *TaTGW6-A1* [79–80]. Using the same comparative genomic approach, Zheng et al. [74] identified the transcript elongation factor *TaTEF-7A* having a strong effect on grain number per spike. Some of the above candidate genes, such as *TaCwi-A1* and *TaGW6-A1*, were located in or near the QTL that were found in the current study and in previous experiments; however, further studies on the characterization of these genes on the tetraploid wheat collection and/or suitable biparental populations are needed to ascertain their implication on the detected QTL.

### Relationships between yield components

The primary components of grain yield potential are GYS and number of spikes per unit area. The latter depends on sowing density and it is strongly affected by environmental factors and agro-technique practices. The sub-components of GYS are the KNS and the TKW. In the current study GYS was significantly positively correlated with KNS and with TKW in all environments and across environments, while the correlations between KNS and TKW achieved at each environment ranged from statistically not significant values to negative and statistically significant coefficients (Table 2). Similar results were previously found in segregating materials and germplasm collections in common wheat [14–16,43,48,81–82]. The predominantly negative correlation between KNS and TKW has been the often observed cause of the increases in KNS partially counterbalanced by the reduction of average grain weight, and vice versa.

**Fig 4:**
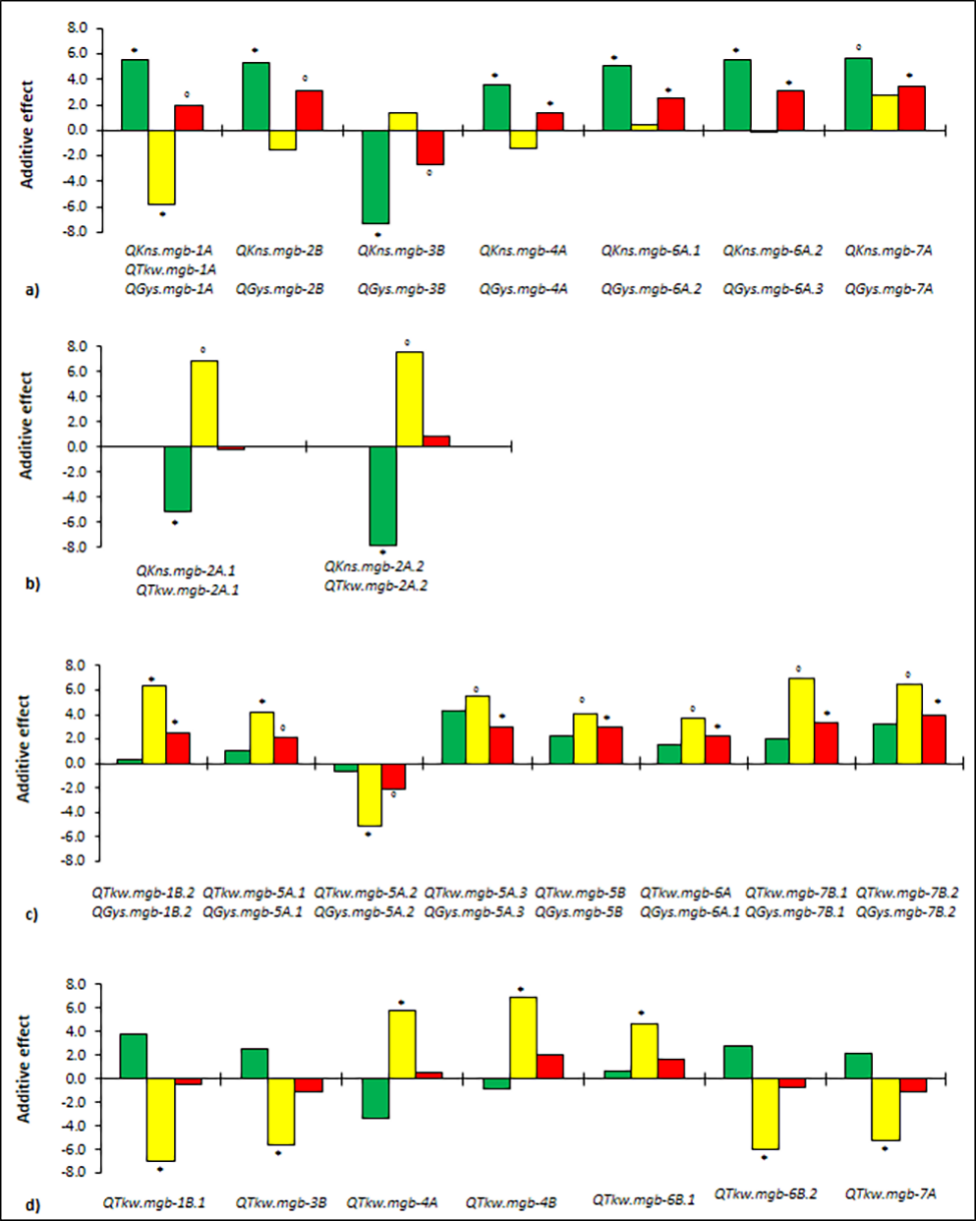
Additive effects of QTL for grain yield per spike (expressed in dg, red bars), number of kernels per spike (green bars) and kernel weight (expressed in mg, yellow bars) identified by GWAS in a tetraploid wheat collection (mean values across seven environments). a) QTL for kernel number per spike associated to QTL for grain yield per spike; b) QTL for kernel number per spike associated to QTL for kernel weight in the opposite direction and without effects on grain yield per spike; c) QTL for kernel weight associated to QTL for grain yield per spike; d) QTL for kernel weight without significant effects on grain yield per spike. The positive or negative additive value refers to the SNP allele with higher frequency. * Significant at -log10(P) ≥3.0, ° significant at 2.0<-log10(P) <3.0.

The QTL detected in the current study could be divided into 4 different groups based on their effects on grain yield per spike (Fig 4):

1. QTL for KNS (*QKns*.*mgb-1A*, *QKns*.*mgb-2B*, *QKns*.*mgb-3B*, *QKns*.*mgb-4A*, *QKns*.*mgb-6A*.*1*, *QKns*.*mgb-6A*.*2*, *QKns*.*mgb-7A*) with a significant effect on GYS. The effects of these QTL on KNS were independent from TKW variation, or the variation in KNS tended to be only partially compensated by not significant variation in TKW in the opposite direction. The QTL *QKns*.*mgb-1A* on chromosome 1A increasing KNS was associated with *QTkw*.*mgb-1A* for a decreasing effect on TKW; the compensating effect was partial and the QTL *QGys*.*mgb-1A* for GYS was found significant at the sub-threshold 2.0>–log_10_(P)>3.0. The genomic regions involved in the quantitative expression of KNS found in the tetraploid wheat collection were compared with the map position of QTL found in previous QTL analyses on the same yield components. The QTL *QKns*.*mgb-1A*, *QKns*.*mgb-2B*, *QKns*.*mgb-6A*.*1*, *QKns*.*mgb-6A*.*2* for increased NKS and GYS were located in or near the QTL regions that were found previously by linkage analysis on biparental populations [15,33,45,57–59]. So far, in our knowledge, *QKns*.*mgb-3B* was not reported in linkage map or association studies before. These seven KNS QTL with direct genetic effects on GYS because of increasing the number of seeds, *i*.*e*. likely directly linked to spikelet fertility, are particular interesting in wheat breeding since they would allow increasing KNS without a related decrease in grain weight.
2. QTL for KNS (*QKns*.*mgb-2A*.*1 and QKns*.*mgb-2A*.*2*) associated to significant QTL with opposite effects on TKW *(QTkw*.*mgb-2A*.*1*, *QTkw*.*mgb-2A*.*2)*. In such cases, the increase of KNS implied a decrease of TKW and the QTL did not contribute to grain yield per spike. *QKns*.*mgb-2A*.*1* was previously detected with a similar compensating effect in the same genomic region of chromosome 2A by Yao et al. [83], Blanco et al. [45] and Jia et al. [60]. These QTL have no actual interest for wheat breeding, but it might be interesting to identify their molecular bases to identify the reasons of the physiological trade-off.
3. QTL for TKW (*QTkw*.*mgb-1B*.*2*, *QTkw*.*mgb-5A*.*1*, *QTkw*.*mgb-5A*.*2*, *QTkw*.*mgb-5A*.*3*, *QTkw*.*mgb-5B*, *QTkw*.*mgb-6A*, *QTkw*.*mgb-7B*.*1*, *QTkw*.*mgb-7B*.*2)* with significant effects on GYS. The effects of these TKW QTL were independent from KNS, or partially compensated by not significant variation in KNS (with opposite effects), resulting in significant increases of GYS. These QTL could represent genes involved in carbohydrate and/or storage protein synthesis with direct consequences on grain weight. Previously described QTL for grain yield associated with grain weight without pleiotropic effects on grain number [15,33,39,43,45,57–59,84–85] were detected in our study near the QTL *QTkw*.*mgb-1B*.*2*, *QTkw*.*mgb-5A*.*1*, *QTkw*.*mgb-5A*.*2*, *QTkw*.*mgb-5A*.*3*, *QTkw*.*mgb-5B*, *QTkw*.*mgb-7B*.*1* and *QTkw*.*mgb-7B*.*2* on chromosomes 1B, 5A, 5B and 7B. *QTkw*.*mgb-6A* was not reported in linkage analysis or in association mapping studies before. These eight TKW QTL are interesting for wheat breeding since they would allow increasing GYS without an associated decrease in grain number.
4. QTL for TKW *(QTkw*.*mgb-1B*.*1*, *QTkw*.*mgb-3B*, *QTkw*.*mgb-4A*, *QTkw*.*mgb-4B*, *QTkw*.*mgb-6B*.*1*, *QTkw*.*mgb-6B*.*2*, *QTkw*.*mgb-7A)* with no effects on GYS. These TKW QTL likely represent genes with pleiotropic effects in the opposite direction on KNS, or genes that are tightly linked to minor QTL for KNS with reduced effects below the detection threshold, thus resulting in complete compensating effects and no influence on GYS. The likelihood to detect a QTL in the QTL mapping is dependent on the ratio between the variance caused by the QTL effect and the total variance of the trait [86]. Minor QTL with limited effects on a trait could have a low variance, therefore such QTL would remain below the statistical detection threshold. The QTL *QTkw*.*mgb-1B*.*1*, *QTkw*.*mgb-4A*, *QTkw*.*mgb-4B* and *QTkw*.*mgb-6B*.*2* were found to be located in the same genomic regions on chromosomes 1B, 4A, 4B and 6B with QTL for TKW detected previously [15,45,57–59,85]. These seven QTL increasing TKW without direct consistent effects on GYS could be important in wheat breeding to improve the marketing value of wheat grain as TKW is directly related to milling quality.

Considering the map position of all detected QTL, except for *QKns*.*mgb-1A*, *QKns*.*mgb-2A*.*1* and *QKns*.*mgb-2A*.*2*, most of the reported QTL for TKW and for KNS was found to be located in different marker intervals, indicating that KNS and TKW are genetically controlled independently each other. The predominantly negative correlation, not always statistically consistent, between KNS and TKW found in the current study and in previous experiments both in segregating materials and wild and cultivated germplasm [14–16,43,48,81–82], could be the result of genetic and/or environment interactions with the availability of photo-assimilates during grain filling [13,18]. However, this widely accepted explanation on the relationships between grain number and grain weight do not excludes a priori the hypothesis of clusters of tightly linked genes with opposite effects on grain yield and/or single genes with pleiotropic effects. Detailed molecular, physiological and biochemical analyses on specific genetic materials, such as near-isogenic lines, could ascertain if the negative correlation is ascribable to pleiotropic effects, tightly linked QTL or compensating effects on the traits.

### Implications of detected QTL for wheat improvement

Grain yield potential is a complex trait with low to intermediate heritability and strong G by E. Phenotypic selection of single plants and/or in single environments in the early segregating generations has not been effective. Molecular markers associated to agronomical valuable QTL can be successfully used in early generation selection in wheat breeding programs for developing improved cultivars [87]. However, QTL analysis aiming to detect useful alleles to be transferred in commercial cultivars by MAS and/or genomic selection schemes should contemplate whether the QTL have pleiotropic effects or are tightly linked to other QTL that will affect negatively the total performance of the genotypes. Moreover, the QTL could play a relevant role in breeding programs if they are stable in different environments and consistently expressed in different genetic backgrounds. In the current study, we detected QTL for two GYS sub-components consistent in at least three environments or in two environments and in the mean across environments. All the detected QTL were also consistent at sub-threshold 2.0>–log10(P)>3.0 in other environments, and all of them had additive effects in the same direction. Most of the QTL for TKW and KNS were found located in different marker intervals, indicating that they are genetically controlled independently by each other. Seven stable QTL for grain number per spike and eight QTL for grain weight were found to be significantly associated to increases of grain yield per spike, indicating that selecting for KNS or TKW or for both yield components could contribute to increase final plant productivity. The identified SNP markers could be usefully deployed in wheat breeding programs for the genetic improvement of grain yield potential. Moreover, QTL accounting for increased TKW without consistent decreasing GYS can be potentially useful to improve the marketing value of wheat grain as TKW is directly related to milling quality.

## Supporting information

S1 Table List of accessions of *T*. *turgidum* subspecies included in the wheat collection. (PDF)

S2 Table Mean squares from the combined analysis of variance for grain yield per spike, thousand kernel weight and kernel per spike across environments in a tetraploid wheat collection evaluated in seven field experiments (accessions evaluated in the seven trials are included in the ANOVA). (PDF)

S3 Table Marker-trait associations for grain yield per spike (GYS), number of kernels per spike (KNS) and thousand kernel weight (TKW) identified by GWAS (model MLM+K) for each of the seven environments (V09, V10, F12, V12, V13, V13_45, V14) and for the mean across environments in a tetraploid wheat collection. (PDF)

S1 Fig Manhattan plot from MLM+K model for the mean values across environments of grain yield per spike (a), thousand kernel weight (b) and number of kernels per spike (c).
(TIF)
