# Supplementary material for: Genetic dissection of the relationships between grain yield components by genome-wide association mapping in a collection of tetraploid wheats
Source: PLoS One. 2018 Jan 11;13(1):e0190162. doi: 10.1371/journal.pone.0190162 (PMC5764242; doi:10.1371/journal.pone.0190162)
Supplement: S1 Table — (PDF) [file pone.0190162.s001.pdf]

S1 Table. List of accessions of *T. turgidum* subspecies included in the wheat collection.

Taxonomic classification	Year of release	Accession	Country	Pedigree
T. turgidum	1915	Cappelli	Italy	Strampelli selection from “Jean Retifah”
ssp. *durum*	1925	Aziziah	Italy	Selected from exotic landraces “Near East”
	1928	Russello	Italy	Selected from landraces Italian
	1930	Timilia	Italy, Sicily	Selected from landraces of South Italy
	1934	Taganrog	Italy	Selected from landraces Russian
	1940	Capeiti-8	Italy	Eiti 6/Cappelli
	1949	Grifoni	Italy	Cappelli/*Triticum aestivum*
	1956	Kyperounda	Marocco	Selected from landraces
	1956	Langdon	United States	Yuma (*T. turgidum* ssp *durum*)/Stewart (*T. turgidum* ssp. *durum*)//Carleton
	1970	Hymera	Italy	B-14/Capeiti-8
	1970	Trinakria	Italy	B-14/Capeiti-8
	1973	Appulo	Italy	Cappelli/Grifoni//Capeiti-8
	1973	Belfuggito	Italy	Conte Morando/3/ *T. turgidum* ssp *dicoccoides* /Duro Australiano// *T. persicum* var *ekinatum* / Duro Australiano
	1973	Lambro	Italy	Conte Morando/3/ *T. turgidum* ssp *dicoccoides* /Duro Australiano// *T. persicum* var *ekinatum* / Duro Australiano
	1974	Creso	Italy	Yaktana-54/Norin 10-B//2*Cappelli-63/3/3*Tehuacan-60/4/Capelli-B144
	1975	Isa	Italy	Sassari 0 130/Patrizio
	1975	Mexicali 75	Mexico	Gerardo-VZ-469/3/Jori (SIB)//ND-61-130/Leeds
	1975	Mida	Italy	Yaktana-54/Norin 10-B//2*Cappelli-63/3/2*Tehuacan-60/4/Capelli-B144
	1975	Polesine	Italy	Forlani/Aziziah
	1975	Valgerardo	Italy	Giorgio-324//Senatore Cappelli/Yuma
	1975	Valnova	Italy	Giorgio-324//Senatore Cappelli/Yuma
	1976	Tito	Italy	Lakota/Castelporziano
	1977	Sansone	Italy	Mara/Cappelli
	1980	Karel	Italy	Mx-198/Maristella
	1980	Produra	United States	Tremes-Molle-Enano/2*Tehuacan-60/3/Zenati-Bouteille/Wells/4/2*Barrigon-Yaqui-Enano/Tehuacan-60/Tacur-Tipo-125-E/2*Tehuacan-60
	1980	Valforte	Italy	VZ-156/Cappelli//Yuma/2*Cappelli
	1981	Berillo	Italy	*Haynaldia villosa* /3*Cappelli/5/Yakatana-54//Norin-10/Brevor/3/ST-464/6/2*Thatcher
	1982	Appio	Italy	Cappelli//Gaviota/Yuma
	1982	Athena	Italy	Ranieri/Jucci
	1982	Latino	Italy	Cappelli/Anhinga//*T. turgidum*
	1982	Messapia	Italy	Mex/Crane (SIB)//Tito
	1983	Arcangelo	Italy	Creso/Appulo
	1983	Lloyd	United States	Cando/Edmore
	1984	Altar84	Mexico	Ruff/Flamingo, Mex//Mexicali 75/3/Shearwater

1984	Duilio	Italy	Cappelli//Anhinga/Flamingo, Mex
1984	Primadur	France	Blondur//2587-8-6/Leeds
1984	Quadruro	Italy	SC-146/ID83-NE
1984	Tresor	Italy	Amber durum/S-22-80
1985	Adamello	Italy	Valforte/Turkis line 7112
1985	Grazia	Italy	Leeds (M 6800127)/Valselva
1986	Ambral	France	D-76018/Valdur
1987	Amedeo	Italy	Maristella/Capeiti
1987	AC Avonlea	Canada	8267-AD2A/DT612
1987	Brindur	France	Crosby/623//Edmore
1987	Neodur	France	184-7/Valdur//Edmore
1988	Agridur	France	Edmore//Cimmyt 303/Chandur
1988	Antas	Italy	Barrigon-Yaqui-Enano/4/Tehuacan-60/3/Yaktana-54//Norin-10/Brevor/5/Tacur-Tipo-125//Tehuacan-60/Ichnusa
1988	Plinio	Italy	Linea D50/Trigo Candeal
1988	Simeto	Italy	Capeit-8/Valnova
1990	Fenix	Italy	Madif/Durum Tunisian//Valgerardo
1990	Ofanto	Italy	Adamello/Appulo
1991	Enduro	Italy	Gaviota/Tehuacan-60//Mexicali-75
1992	Cirillo	Italy	Jucci/Polesine//Creso/Montanari
1992	Cosmodur	France	Natural hybridization of D881 line
1992	Dauno	Italy	Gediz-75/Flamingo, Mex//Teal, Mex
1992	Doral	France	INRA-164-1-27/IDSN-45
1992	Exeldur	France	Valdur/Regal
1992	Fauno	Italy	Gediz-75/Flamingo, Mex//Teal, Mex
1992	Gianni	Italy	Multiple cross among durum wheat cultivar
1992	Granizo	Spain	Yel S/Shaw S
1992	Parsifal	France	INRA 92-1/D81028
1992	Zenit	Italy	Valriccardo/Vic
1993	Italo	Italy	Complex cross between Italian and Turkish genotypes
1993	Kronos	United States	APB MSFRS POP Sel (D03-12)
1994	Ceedur	France	Mondur/2587.8.6//Edmore/Chandur
1995	Arcobaleno	Italy/Spain	Chen/Altar 84
1995	Ares	Italy	Lira/Vic
1995	Colosseo	Italy	Mutant Mexa/Creso
1995	Fortore	Italy	Capeiti-8/Valforte
1995	Platani	Italy	Valnova/Capeiti-8
1995	Preco	Italy	Edmore/WPB881//Selected line 3
1995	Saadi	France	IDSM72-3/711.8

1996	Bronte	Italy	Berillo/Latino
1996	Ciccio	Italy	F6 Appulo/Valnova//F5 Valforte/Patrizio
1996	Durfort	France	Selected from REVA population
1996	Iride	Italy	Altar 84/Ares
1996	Nefer	France	164/Keops
1996	Rusticano	Italy	n.a.
1996	San Carlo	Italy	Grazia/Degamit
1996	Svevo	Italy	CIMMYT's selection/Zenit
1996	Vitromax	Italy/Spain	Turchia77/3/Jori/Anhunga//Flamingo, Mex
1997	Varano	Italy	Capeiti-8/Creso//Creso/3/Valforte/Trinakria
1998	AC Navigator	Canada	Kyle/WB 881
1998	Baio	Italy	Duilio/F21//G76
1998	Cannizzo	Italy	F5 Capeiti/Valnova// F5 Patrizio/Valforte
1998	Claudio	Italy	CIMMYT's selection 35/Durango//IS1938/Grazia
1998	Martino	Italy	Appulo/Produra
1998	Provenzal	Italy	CIMMYT's selection (ATO’S CII3EIPUSOA 580)
1999	Giotto	Italy	W.A 6518-2/GA547
1999	Meridiano	Italy	Simeto/WB 881/Duilio/F21
1999	Orobel	Italy	Composite INRA/Gil Ble Dur
1999	Quadrato	Italy	Creso/Trinakria
1999	Vesuvio	Italy	Ofanto/Simeto
2001	Avispa	Italy	n.a.
2001	Fiore	Italy	Derived from CIMMYT's selection
2001	Tiziana	Italy	Peleo/Neodur
2002	Duetto	Italy	1485 x 83.74
2002	Dylan	Italy	Neodur/Ulisse
2002	Grecale	Italy	S2/WB 881//Plinio/F22
2002	Normanno	Italy	Simeto/F22//L35
2002	Virgilio	France	Acalou/Shoula
2003	Ancomarzio	Italy	Stotka//Altar84/Ald
2003	Casanova	Italy	Flavio/Syene//Duilio
2003	Chiara	Italy	Arcangelo/Fortore
2003	Latinur	France	n.a.
2003	Vendetta	Italy	Creso/Ofanto
2004	L092	United States	1A. 1D/Len//Langdon/3/2*Renville
2004	L252	United States	1A. 1D/Len//Langdon/3/2*Renville
2004	Maestrale	Italy	Iride/Svevo
2004	Orfeo	Italy	Creso/Simeto

	2004	S99B34	United States	1A. 1D/Len//Langdon/3/2*Renville
	2004	Saragolla	Italy	Iride/Line SPB 0114
	2005	Ariosto	Italy	Karim/GA7-X3//Duilio
	2005	Arnacoris	Italy	n.a.
	2005	Canyon	Italy	n.a.
	2005	Imhotep	Italy	n.a.
	2005	PR22D89	Italy	Ofanto/Duilio//Ixos
	2005	Strongfield	Canada	AC-Avonlea/DT-665
	2006	Alemanno	Italy	Cappelli/Duilio
	2006	Ciclope	Italy	Trinakria/Berillo//Valnova/Trinakria
	2006	K26	Italy	Spontaneuos mutant derived from Simeto/Colosseo
	2006	UC1113	Canada	Kingfisher //Rossia/BD-1419/3/Mexi/CP/4/Waha/5/Yavaros-79
	2007	Neolatino	Italy	Latino/Trinakria//MG1433/4/Latino
	2007	Liberdur	France	Nefer/acalou//ga7-b14
	2007	Isildur	France	n.a.
	2009	Aureo	Italy	Kofa x Svevo
	-	5-BIL42	Italy	Breeding line derived from Latino/MG29896
	-	PC32	Italy	Breeding line derived from F6 Latino/Primadur
	n.a.	Barcarol	Italy	n.a.
	n.a.	Pedroso	Spain	n.a.
	n.a.	Sharm 5	Syria	Selected from landraces Syrian
	n.a.	West Bread 881	United States	Complex cross of Ward, Wells, Cando, Waskana, Mexicali 75
T. turgidum	K cer	Egypt		
ssp. *turanicum*	Cltr-11390	United State		
	PI 68287	Azerbaijan		
	PI 113393	Iraq		
	PI 167481	Turkey, Denizli		
	PI 191599	Marocco, Rabat-Sale		
	PI 192641	Morocco		
	PI 254206	Iran		
	PI 278350	Italy		
	PI 290530	Hungary, Pest		
	PI 306665	France, Herault		
	PI 576854	Turkey, Diyarbakir		
	PI 623656	Iran, West Azerbaijan		
	PI 624429	Iran, Bakhtaran		
	PI 127106	Afghanistan, Faryab		
	PI 67343	Australia, Victoria		

	PI 192658	Morocco
	PI 184526	Portugal
	PI 352514	Azerbaijan
	PI 362067	Romania, Brasov
T. turgidum	PI 56263	Portugal, Lisboa
ssp. *turgidum*	PI 134946	Portugal, Lisboa
	PI 157983	Italy, Sicily
	PI 157985	Italy, Sicily
	PI 173503	Turkey, Artvin
	PI 185723	Portugal, Leira
	PI 191104	Spain
	PI 191145	Spain, Baleares
	PI 191203	Spain
	PI 286075	Poland
	PI 221423	Portugal
	PI 352544	Switzerland, Vaud
	PI 290522	Germany
	PI 290526	Hungary, Pest
	PI 341391	Turkey, Burdur
	PI 352538	United Kingdom
	PI 352541	Germany
	PI 352542	France
	PI 352543	France
T. turgidum	PI 266846	United Kingdom, England
ssp. *polonicum*	PI 278647	United Kingdom, England
	PI 289606	United Kingdom, England
	PI 330554	United Kingdom, England
	PI 330555	United Kingdom, England
	PI 349051	Georgia
	PI 352487	Germany, Saxony-Anhalt
	PI 352488	Italy
	PI 352489	Cyprus
	PI 366117	Egypt, Sinai
	PI 387479	Ethiopia
	PI 566593	United States
	PI 208911	Iraq
	PI 210845	Iran
	PI 223171	Jordan

	PI 272564	Hungary, Pest
	PI 286547	Ecuador
	PI 290512	Portugal
	PI 306549	Romania
T. turgidum	Citr 7665	Russian Federation
ssp. *carthlicum*	PI 70738	Iraq
	PI 94755	Georgia
	PI 115816	Georgia
	PI 283888	Iran
	PI 341800	Russian Federation, Dagest
	PI 499972	Georgia
	PI 532501	Former Soviet Union
	PI 572849	Georgia
	PI 573182	Turkey, Kars
	PI 585017	Georgia
	PI 585018	Georgia
T. turgidum	Farvento	Italy
ssp. *dicoccum*	Lucanica	Italy
	Molise selezion	Italy
	ISC Foggia 152	Iran
	ISC Foggia 159	Morocco
	ISC Foggia 161	United Kingdom
	ISC Foggia 171	Ethiopia
	ISC Foggia 175	Hungary
	MG 5350	Ethiopia
	MG 4387	United Kingdom
	MG 5416/1	Iran
	MG 5471/1	Spain
	MG 5473	Spain
	MG 15516/1	Syria
	MG 5344/1	Ethiopia
	MG 5293/1	Italy
	MG 5323	n.a.
	MG 3521	n.a.
	MG 5300/1	n.a.
T. turgidum	PI 346783	Hungary, Pest
ssp. *dicoccoides*	PI 343446	Israel
	PI 481539	Israel

PI 352323	Asia Minor
PI 352324	Lebanon
PI 355459	Armenia
PI 470944	Syria, Al Qunaytirah
PI 470945	Syria, Al Qunaytirah
MG 4343	n.a.
MG 4328/61	n.a.
MG 5444/235	n.a.
MG 4330/66	n.a.

n.a. not available

CItr and PI number indicate the accession number in USDA National Small Grains Collection, Aberdeen, Idaho, USA.

MG number indicate the accession number in CNR Institute of Plants Genetics, Bari, Italy.

ISC Foggia number indicate the accession number in CRA-CER Cereal Research Centre, Foggia, Italy.
