# Supplementary material for: Genetic dissection of the relationships between grain yield components by genome-wide association mapping in a collection of tetraploid wheats
Source: PLoS One. 2018 Jan 11;13(1):e0190162. doi: 10.1371/journal.pone.0190162 (PMC5764242; doi:10.1371/journal.pone.0190162)
Supplement: S2 Table — (PDF) [file pone.0190162.s002.pdf]

S2 Table. Mean squares from the combined analysis of variance for grain yield per spke, thousand kernel weight and kernel per spike across environments in a tetraploid wheat collection evaluated in seven field experiments (accessions evaluated in the seven trials are included in the ANOVA).

Source of variation	df	Grain yield per spike	Thousand kernel weight	Kernel per spike
Enviroment (E)	6	40.675***	15511.346***	9257.717***
Blocks/Environment	14	1.273	88.042	331.146
Genotype (G)	199	3.777***	1770.589***	922.288***
G x E	1194	0.327***	54.930***	91.662***
Error	2786	0.060	6.784	15.485

*** Significant at the $P \leq 0.001$ level.
