# Supplementary material for: Genetic dissection of the relationships between grain yield components by genome-wide association mapping in a collection of tetraploid wheats
Source: PLoS One. 2018 Jan 11;13(1):e0190162. doi: 10.1371/journal.pone.0190162 (PMC5764242; doi:10.1371/journal.pone.0190162)
Supplement: S3 Table — (PDF) [file pone.0190162.s003.pdf]

S3 Table. Marker-trait associations for grain yield per spike (GYS), number of kernels per spike (KNS) and thousand kernel weight (TKW) identified by GWAS (model MLM+K) for each of the seven environments (V09, V10, F12, V12, V13, V13_45, V14) and for the mean across environments in a tetraploid wheat collection.

QTL	Trait	Marker	Chrom	Map position (cM)	SNP	SNP Frequency	Environments																							
							V09			V10			F12			V12			V13			G13			V14			Mean		
							P	Add	R^2(%)	P	Add	R^2(%)	P	Add	R^2(%)	P	Add	R^2(%)	P	Add	R^2(%)	P	Add	R^2(%)	P	Add	R^2(%)	P	Add	R^2(%)
QGys.mgb-1A°	GYS	IWB57483	1A	50.4	A/G	148/65	1.6	0.2	2.4	0.1	0.0	0.1	**3.2**	0.4	5.5	0.5	0.1	0.4	0.0	0.0	0.0	0.2	0.1	0.1	**2.0**	0.3	3.0	**2.1**	0.2	3.1
*QKns.mgb-1A**	KNS	IWB57483	1A	50.4	A/G	148/65	**2.6**	5.1	4.4	1.3	2.9	1.8	**4.8**	9.5	9.0	**2.2**	5.1	3.6	1.6	3.7	2.2	1.0	2.8	1.2	**4.1**	8.0	7.5	**4.5**	5.6	7.8
*QTkw.mgb-1A**	TKW	IWB48626	1A	50.4	C/T	170/50	**2.7**	-5.9	4.4	**2.1**	-5.4	3.4	1.8	-4.7	2.6	**2.0**	-4.7	3.0	**4.2**	-8.4	7.1	**3.6**	-8.1	5.8	1.6	-4.8	2.3	**3.3**	-5.9	5.4
	GYS	IWB46333	1B	53.9-57.6	A/G	187/28	0.0	0.0	0.0	0.3	-0.1	0.2	0.4	-0.1	0.4	0.7	0.1	0.7	0.5	-0.1	0.4	0.0	0.0	0.0	0.3	-0.1	0.3	0.2	-0.1	0.1
	KNS	IWB46333	1B	53.9-57.6	A/G	187/28	**2.1**	5.5	3.3	1.6	4.2	2.4	0.4	1.9	0.3	1.2	3.3	1.6	1.3	4.1	1.8	1.2	3.9	1.6	1.2	4.4	1.6	1.7	3.8	2.6
*QTkw.mgb-1B.1**	TKW	IWB46333	1B	53.9-57.6	A/G	187/28	**2.6**	-6.4	4.2	**4.4**	-9.6	8.3	1.9	-5.9	2.9	**2.3**	-4.3	3.5	**2.7**	-7.5	4.2	**2.3**	-6.6	2.9	**3.6**	-9.2	6.3	**3.4**	-7.0	5.6
*QGys.mgb-1B**	GYS	IWB66475	1B	152-155.3	A/G	188/29	0.4	0.1	0.3	**2.8**	0.3	4.7	1.8	0.4	2.6	0.7	0.2	0.7	**3.9**	0.5	6.8	**2.4**	0.4	3.7	**3.9**	0.5	7.0	**3.4**	0.2	5.5
	KNS	IWB66475	1B	152-155.3	A/G	188/29	1.4	4.6	2.0	0.4	1.4	0.3	0.3	1.7	0.2	0.8	3.0	0.9	0.8	3.2	0.9	0.2	1.1	0.1	0.4	2.3	0.3	0.1	0.3	0.0
*QTkw.mgb-1B.2**	TKW	IWB66475	1B	152-155.3	A/G	188/29	1.2	4.1	1.7	1.6	5.1	2.3	**2.3**	6.3	3.6	1.1	3.7	1.4	**5.4**	10.8	9.8	**4.3**	10.3	7.4	0.5	2.2	0.4	**3.2**	6.3	5.0
	GYS	IWB27389	2A	113.6-114.7	A/G	167/45	0.4	-0.1	0.3	0.7	0.1	0.8	0.3	-0.1	0.2	0.3	0.1	0.2	0.1	0.0	0.0	0.1	0.0	0.0	0.0	0.0	0.0	0.1	0.0	0.0
*QKns.mgb-2A.1**	KNS	IWB46763	2A	113.6-114.7	A/G	167/49	**3.7**	-8.0	6.7	1.8	-3.9	2.7	**4.7**	-12.4	8.6	1.0	-3.2	1.3	**2.8**	-5.1	4.5	**2.4**	-5.5	3.8	**2.6**	-7.5	4.2	**4.2**	-5.2	7.4
QTkw.mgb-2A.1°	TKW	IWB27389	2A	113.6-114.7	A/G	167/45	**2.0**	5.1	3.3	**5.6**	9.9	10.7	**2.7**	6.3	4.5	**2.6**	5.6	4.3	**2.6**	6.5	4.1	**2.9**	7.3	4.6	**2.0**	5.9	3.3	**4.1**	6.9	7.3
	GYS	IWB1937	2A	181.2	T/C	184/31	0.5	0.2	0.4	0.3	0.1	0.2	1.2	0.4	1.5	0.2	0.1	0.1	0.8	0.2	0.9	0.7	0.2	0.8	0.3	0.1	0.2	0.3	0.1	0.3
*QKns.mgb-2A.2**	KNS	IWB1937	2A	181.2	T/C	184/31	0.7	-3.6	0.8	0.8	-3.7	1.0	**3.9**	-13.7	6.9	1.0	-3.5	1.2	**4.1**	-11.6	7.5	**2.7**	-9.2	4.5	1.2	-6.4	1.7	**3.6**	-7.9	6.3
QTkw.mgb-2A.2°	TKW	IWB1937	2A	181.2	T/C	184/31	**2.4**	8.2	3.8	1.4	6.3	1.9	1.6	6.9	2.3	1.6	6.1	2.3	**2.8**	10.2	4.3	**2.1**	9.0	3.0	1.2	5.9	1.5	**2.5**	7.6	3.7
QGys.mgb-2B°	GYS	IWB2285	2B	70.4-76.6	T/C	161/59	0.1	0.0	0.1	0.9	0.1	1.0	0.4	0.1	0.3	0.5	0.1	0.5	0.5	0.1	0.4	**2.3**	0.4	3.5	**3.3**	0.3	5.7	**3.0**	0.3	4.8
*QKns.mgb-2B**	KNS	IWB2285	2B	70.4-76.7	T/C	161/59	0.4	1.3	0.3	**3.5**	5.3	6.1	**2.2**	5.9	3.5	1.5	3.8	2.2	**2.3**	4.6	3.6	**3.4**	8.3	5.7	**3.3**	9.4	5.6	**2.7**	5.3	4.1
	TKW	IWB2285	2B	70.4-76.8	T/C	161/59	0.1	-0.4	0.0	0.8	-2.5	0.9	1.3	-3.6	1.8	0.5	-1.5	0.4	0.7	-2.3	0.7	0.6	-2.0	0.5	0.5	-1.9	0.5	0.5	-1.5	0.4
	GYS	IWB47344	3B	148.4	C/T	168/48	1.1	-0.2	1.5	**2.7**	-0.3	4.5	0.0	0.0	0.0	0.0	0.0	0.0	0.1	0.0	0.0	0.1	0.0	0.0	**2.3**	-0.3	3.7	0.9	-0.1	1.0
	KNS	IWB47344	3B	148.4	C/T	168/48	0.1	-0.7	0.1	0.3	1.0	0.2	1.6	5.0	2.3	0.5	1.6	0.4	1.2	3.3	1.6	0.7	2.2	0.7	0.2	-0.9	0.1	1.3	2.5	1.6
*QTkw.mgb-3B**	TKW	IWB47344	3B	148.4	C/T	168/48	1.4	-3.9	2.0	**5.5**	-9.4	10.3	**2.2**	-5.2	3.5	**3.0**	-5.3	5.1	1.7	-4.5	2.3	**2.6**	-4.8	3.9	**4.1**	-8.1	7.1	**3.4**	-5.6	5.5
QGys.mgb-3B°	GYS	IWB55725	3B	186.6	A/G	198/19	0.1	0.0	0.1	0.2	-0.1	0.1	0.0	0.0	0.0	0.7	-0.2	0.7	**3.3**	-0.5	5.4	**4.1**	-0.6	7.2	1.0	-0.3	1.3	**2.0**	-0.3	2.8
*QKns.mgb-3B**	KNS	IWB55725	3B	186.6	A/G	198/19	**2.0**	-6.68	3.3	**2.6**	-6.1	4.2	0.6	-3.2	0.5	0.9	-3.8	1.0	**4.9**	-11.7	8.9	**5.8**	-12.9	10.8	**2.1**	-7.6	3.2	**3.9**	-7.4	6.4
	TKW	IWB55725	3B	186.6	A/G	198/19	0.4	2.1	0.3	0.7	3.4	0.7	0.9	4.1	1.1	0.2	-1.2	0.1	0.0	0.2	0.0	0.6	3.1	0.6	0.1	0.8	0.0	0.3	1.3	0.2
	GYS	IWB55207	4A	61.2	C/A	184/30	1.5	0.2	2.1	0.9	0.2	1.1	0.3	0.1	0.2	0.3	0.1	0.2	0.4	-0.1	0.3	0.4	0.1	0.3	0.0	0.0	0.0	0.3	0.1	0.2
	KNS	IWB55207	4A	61.2	C/A	184/30	0.1	-0.7	0.1	0.8	-2.2	0.9	0.9	-3.7	1.2	0.1	-0.6	0.1	**2.4**	-5.9	3.8	0.5	-1.9	0.4	0.9	-3.5	1.1	1.6	-3.4	2.2
*QTkw.mgb-4A**	TKW	IWB55207	4A	61.2	C/A	184/30	**3.2**	7.3	5.5	**3.8**	8.5	6.8	**3.3**	7.6	5.6	0.8	2.7	0.8	**2.2**	6.1	3.2	**2.7**	6.6	4.2	1.7	5.1	2.3	**2.9**	5.8	4.5
*QGys.mgb-4A**	GYS	IWB71701	4A	167.6-170.9	C/T	146/70	**3.8**	0.3	6.7	1.5	0.2	2.2	**3.3**	0.5	5.6	1.2	0.1	1.6	1.0	0.1	1.2	0.2	0.0	0.1	**3.0**	0.3	5.3	**2.0**	0.1	2.8
*QKns.mgb-4A**	KNS	IWB71701	4A	167.6-170.9	C/T	146/70	**5.9**	6.4	11.6	**2.4**	4.0	3.9	**2.1**	5.8	3.3	**2.1**	3.3	3.3	**2.3**	3.5	3.6	0.7	1.7	0.8	**3.2**	5.2	5.5	**3.6**	3.6	6.4
	TKW	IWB71701	4A	167.6-170.9	C/T	146/70	0.1	0.2	0.0	0.5	2.1	0.4	1.9	4.6	2.9	0.2	-0.7	0.1	1.3	-2.8	1.6	1.6	-3.4	2.1	0.1	0.3	0.0	0.6	-1.4	0.6
	GYS	IWB4448	4B	103.6-109.1	T/C	192/26	0.5	0.1	0.4	1.1	0.2	1.5	1.2	0.3	1.6	0.7	0.1	0.7	0.8	0.1	0.8	0.5	0.1	0.4	0.1	0.0	0.0	1.6	0.2	2.2
	KNS	IWB4448	4B	103.6-109.2	T/C	192/26	0.6	-2.2	0.6	0.8	-2.3	0.9	0.1	0.9	0.1	0.3	-1.2	0.2	0.6	-2.2	0.7	0.9	-2.7	1.0	0.5	-2.8	0.4	0.2	-0.9	0.1
*QTkw.mgb-4B**	TKW	IWB4448	4B	103.6-109.3	T/C	192/26	1.8	4.5	2.7	**3.8**	9.1	6.8	**2.4**	6.0	3.8	**2.8**	5.5	4.6	**2.6**	5.9	3.9	**3.8**	7.3	6.2	1.7	5.3	2.4	**3.4**	7.0	5.5
QGys.mgb-5A.1°	GYS	IWB35357	5A	14.3-23.2	G/C	193/27	**4.0**	0.4	7.1	1.7	0.2	2.6	1.9	0.3	2.8	1.8	0.2	2.6	**2.7**	0.2	4.3	1.1	0.1	1.5	0.5	0.1	0.5	**3.6**	0.2	6.1
	KNS	IWB43705	5A	14.3-23.2	A/G	156/56	0.1	0.3	0.0	0.2	0.5	0.1	0.2	0.8	0.1	0.3	0.9	0.2	0.4	1.1	0.4	0.1	-0.4	0.1	0.0	-0.1	0.0	0.5	1.1	0.5
*QTkw.mgb-5A.1**	TKW	IWB43705	5A	14.3-23.2	A/G	156/56	**2.8**	4.3	5.0	**2.6**	4.6	4.4	**3.0**	4.8	5.1	**4.2**	5.5	7.6	**2.1**	3.8	3.1	**2.1**	4.0	3.1	1.1	2.7	1.4	**3.3**	4.2	5.5
QGys.mgb-5A.2°	GYS	IWB71420	5A	70.8-73.6	C/T	183/34	**2.3**	-0.3	3.6	0.7	-0.1	0.7	0.3	-0.1	0.2	0.6	-0.1	0.6	1.4	-0.2	2.0	0.8	-0.1	0.9	**3.4**	-0.4	6.1	**2.7**	-0.2	4.1
	KNS	IWB11637	5A	70.8-73.6	G/A	153/58	0.3	-1.1	0.2	0.8	1.9	1.0	0.2	1.0	0.1	0.0	-0.1	0.0	0.0	0.0	0.0	0.6	1.6	0.6	0.9	-2.7	1.1	0.2	-0.6	0.1
*QTkw.mgb-5A.2**	TKW	IWB11637	5A	70.8-73.6	G/A	153/58	**2.4**	-4.6	3.8	**2.3**	-4.6	3.6	1.6	-3.8	2.4	**3.2**	-4.9	5.4	**2.6**	-5.1	4.1	**3.9**	-6.9	6.6	**3.2**	-6.5	5.4	**3.8**	-5.1	6.3

*QGys.mgb-5A.3**	GYS	IWB12854	5A	111.5-114.3	T/C	190/28	**3.6**	0.5	6.5	1.3	0.2	1.7	1.6	0.3	2.3	**3.8**	0.4	6.4	0.9	0.2	1.0	**2.7**	0.4	4.3	1.5	0.3	2.1	**3.4**	0.3	5.5
	KNS	IWB11972	5A	111.5-114.3	C/T	194/24	**3.4**	8.3	6.0	0.4	1.6	0.3	0.2	1.7	0.1	1.6	4.7	2.4	1.3	4.5	1.7	1.8	5.8	2.7	0.2	1.5	0.1	1.9	4.3	2.7
QTkw.mgb-5A.3°	TKW	IWB12854	5A	111.5-114.3	T/C	190/28	1.1	4.3	1.5	1.3	4.3	1.7	**4.1**	8.6	7.3	**2.9**	6.3	4.7	1.6	5.1	2.1	0.2	1.3	0.1	**2.9**	8.0	4.6	**2.5**	5.5	3.7
*QGys.mgb-5B**	GYS	IWB66934	5B	82.0	T/C	149/70	**4.4**	0.4	8.0	**3.0**	0.3	5.0	**4.1**	0.4	7.3	1.5	0.2	2.1	**2.5**	0.3	3.8	**3.1**	0.3	4.9	**3.5**	0.4	6.1	**5.4**	0.3	9.4
	KNS	IWB66934	5B	82.0	T/C	149/70	1.6	3.5	2.4	1.0	2.1	1.3	0.9	2.7	1.0	0.6	1.7	0.6	0.3	1.0	0.2	0.5	1.5	0.5	1.3	3.6	1.8	1.3	2.3	1.7
QTkw.mgb-5B°	TKW	IWB66934	5B	82.0	T/C	149/70	**2.7**	5.0	4.4	**2.0**	4.2	3.0	**3.2**	5.5	5.4	1.3	2.8	1.6	1.5	3.6	2.0	**2.5**	5.4	3.8	**2.3**	5.4	3.8	**2.5**	4.1	3.9
*QGys.mgb-6A.1**	GYS	IWB23313	6A	0.0-0.1	C/A	176/38	**3.9**	0.3	7.0	**3.8**	0.3	6.7	0.7	0.2	0.8	1.6	0.2	2.2	**2.0**	0.2	3.0	**2.4**	0.3	3.8	1.0	0.2	1.3	**3.3**	0.2	5.2
	KNS	IWB23313	6A	0.0-0.1	C/A	176/38	1.3	3.1	1.8	1.4	2.8	2.0	0.1	-0.7	0.1	0.1	0.3	0.0	0.3	0.9	0.2	0.6	1.8	0.7	0.4	1.4	0.3	0.7	1.5	0.7
QTkw.mgb-6A°	TKW	IWB23313	6A	0.0-0.1	C/A	176/38	**2.6**	4.8	4.6	1.2	3.2	1.7	**2.4**	4.9	3.9	1.4	3.2	2.0	1.8	4.0	2.4	**2.0**	4.7	2.9	1.2	3.4	1.5	**2.1**	3.7	3.1
*QGys.mgb-6A.2**	GYS	IWB24036	6A	13.9	C/A	184/28	1.1	0.2	1.5	**2.1**	0.3	3.3	**2.2**	0.3	3.4	**3.3**	0.3	5.7	**2.3**	0.3	3.6	**3.7**	0.4	6.3	0.3	0.1	0.2	**3.3**	0.2	5.4
*QKns.mgb-6A.1**	KNS	IWB24036	6A	13.9	C/A	184/28	1.5	3.8	2.2	**3.2**	4.8	5.4	**2.7**	6.5	4.4	**3.5**	5.8	6.1	**2.8**	5.4	4.6	**3.5**	6.1	5.9	0.7	2.4	0.7	**4.0**	5.0	6.5
	TKW	IWB24036	6A	13.9	C/A	184/28	0.5	1.8	0.4	0.4	1.6	0.3	0.4	1.4	0.3	0.3	-1.1	0.2	0.1	-0.5	0.0	0.0	-0.2	0.0	0.4	-1.8	0.4	0.1	0.4	0.0
*QGys.mgb-6A.3**	GYS	IWB38787	6A	52.6	T/C	187/25	0.7	0.2	0.7	1.8	0.3	2.8	1.4	0.3	2.0	0.1	0.0	0.0	**3.6**	0.5	6.3	**3.2**	0.5	5.4	0.8	0.2	0.9	**3.1**	0.3	4.8
*QKns.mgb-6A.2**	KNS	IWB38787	6A	52.6	T/C	187/25	0.7	2.7	0.8	**2.6**	5.9	4.4	1.6	6.3	2.4	0.5	2.0	0.4	**3.6**	7.8	6.4	**3.1**	8.5	5.0	1.4	5.0	1.9	**3.2**	5.5	5.3
	TKW	IWB38787	6A	52.6	T/C	187/25	0.3	1.4	0.2	0.1	-0.3	0.0	0.1	0.9	0.1	0.1	-0.8	0.1	0.0	0.0	0.0	1.1	4.5	1.2	0.1	-0.7	0.0	0.0	-0.1	0.0
	GYS	IWB45190	6B	38.2	G/A	183/22	**4.6**	0.5	9.0	0.9	0.2	1.0	0.7	0.2	0.7	1.6	0.2	2.5	1.3	0.2	1.7	0.1	0.0	0.0	0.1	0.0	0.0	1.6	0.2	2.3
	KNS	IWB45190	6B	38.2	G/A	183/22	1.9	4.7	3.2	0.1	0.3	0.0	1.1	-4.4	1.5	0.3	1.2	0.2	0.2	0.9	0.1	1.1	-2.9	1.5	0.7	-2.5	0.8	0.2	0.7	0.1
*QTkw.mgb-6B.1**	TKW	IWB45190	6B	38.2	G/A	183/22	**3.3**	6.6	5.9	1.2	3.7	1.5	**3.3**	7.3	5.7	**4.2**	7.1	7.9	1.7	4.3	2.2	1.2	3.5	1.5	0.9	3.0	1.0	**2.6**	4.7	3.9
	GYS	IWB34014	6B	105.5	T/C	203/17	0.2	-0.1	0.1	0.3	-0.1	0.2	0.6	-0.2	0.6	0.5	-0.1	0.5	1.2	-0.2	1.5	1.7	-0.3	2.3	0.2	0.1	0.1	0.5	-0.1	0.4
	KNS	IWB34014	6B	105.5	T/C	203/17	1.2	3.7	1.6	0.9	2.6	1.1	0.5	2.3	0.5	0.5	2.0	0.5	0.6	2.2	0.6	0.1	0.7	0.1	1.4	4.6	1.9	1.3	2.8	1.6
*QTkw.mgb-6B.2**	TKW	IWB34014	6B	105.5	T/C	203/17	**2.6**	-5.9	4.2	**2.1**	-5.4	3.2	**3.3**	-7.4	5.7	1.9	-4.6	2.7	**3.1**	-6.9	4.8	**3.3**	-7.7	5.3	0.8	-3.1	0.9	**3.5**	-5.9	5.5
*QGys.mgb-7A**	GYS	IWB21281	7A	134.1	T/G	188/27	**3.4**	0.5	5.9	0.1	0.1	0.1	1.7	0.4	2.4	1.6	0.3	2.3	**4.0**	0.6	6.9	1.4	0.3	2.0	0.8	0.3	1.0	**3.6**	0.3	6.0
QKns.mgb-7A°	KNS	IWB21281	7A	134.1	T/G	188/27	**2.8**	7.9	4.7	0.2	0.9	0.1	0.6	3.1	0.6	1.9	5.2	3.0	**2.6**	7.7	4.1	0.5	2.7	0.5	1.1	5.2	1.4	**2.9**	5.6	4.7
	TKW	IWB21281	7A	134.1	T/G	188/27	1.2	4.9	1.7	0.4	1.9	0.3	1.1	4.2	1.4	0.8	3.1	0.9	0.4	2.0	0.4	0.4	2.1	0.3	0.5	2.7	0.4	0.8	2.7	0.8
	GYS	IWB33997	7A	189.5	A/G	176/37	0.8	-0.1	1.0	0.6	-0.1	0.6	1.0	-0.2	1.3	0.1	0.0	0.0	0.6	-0.1	0.5	1.0	-0.1	1.2	1.6	-0.2	2.3	1.1	-0.1	1.3
	KNS	IWB33997	7A	189.5	A/G	176/37	0.6	1.7	0.6	1.7	3.0	2.5	0.6	2.2	0.7	1.1	2.7	1.4	0.9	2.2	1.0	0.2	0.8	0.1	0.2	0.9	0.1	1.2	2.2	1.5
*QTkw.mgb-7A**	TKW	IWB33997	7A	189.5	A/G	176/37	**2.4**	-4.4	3.9	**3.5**	-6.0	6.3	**3.8**	-6.3	6.8	**3.5**	-5.3	5.9	1.9	-4.2	2.6	**2.7**	-5.3	4.3	**3.5**	-6.6	5.8	**3.9**	-5.3	6.4
*QGys.mgb-7B.1**	GYS	IWB12937	7B	65.5-73.4	C/T	197/22	**3.0**	0.4	5.0	**3.7**	0.4	6.3	**2.0**	0.5	3.0	**3.8**	0.5	6.2	1.0	0.3	1.3	0.7	0.2	0.8	0.1	0.0	0.0	**2.4**	0.3	3.5
	KNS	IWB12937	7B	65.5-73.4	C/T	197/22	0.5	3.0	0.5	1.9	4.3	2.8	0.9	5.2	1.1	**2.4**	6.0	3.8	0.2	1.4	0.1	0.3	2.0	0.2	0.2	-1.9	0.1	0.5	2.0	0.4
QTkw.mgb-7B.1°	TKW	IWB12937	7B	65.5-73.4	C/T	197/22	**2.3**	8.4	3.7	**2.0**	8.5	3.1	1.9	8.0	2.8	**2.3**	7.6	3.5	1.7	7.5	2.3	0.9	5.3	1.0	1.1	6.0	1.4	**2.1**	7.0	3.0
*QGys.mgb-7B.2**	GYS	IWB60564	7B	104.7	A/G	179/39	**2.3**	0.4	3.7	**4.4**	0.6	7.8	**3.6**	0.7	6.3	**3.2**	0.6	5.4	1.9	0.4	2.9	1.3	0.3	1.7	0.9	0.3	1.1	**3.7**	0.4	6.0
	KNS	IWB60564	7B	104.7	A/G	179/39	0.5	2.6	0.5	1.7	4.6	2.4	1.6	7.2	2.3	**2.5**	8.3	4.1	0.6	2.7	0.5	0.3	1.8	0.2	0.2	1.4	0.1	1.0	3.2	1.2
QTkw.mgb-7B.2°	TKW	IWB60564	7B	104.7	A/G	179/39	**2.2**	7.2	3.4	**2.9**	9.4	4.9	**2.4**	7.5	3.8	**2.2**	7.8	3.5	1.5	6.3	2.0	1.6	6.9	2.2	1.1	5.3	1.4	**2.3**	6.5	3.4

* QTL significant at -log10(*P*)≥3.0 in at least three environments or two environments and in the mean across environments (P value in bold and in red).

° Suggestive QTL significant at 2.0≤-log10(*P*)≤3.0 in at least three environments or two environments and in the mean across environments (P value in bold and in pink). .

Chromosome and map position from Maccaferri et al. (2014)

Phenotypic variation (R^2%) explained by the QTL.

Add = Additive effect of the QTL. The + or - sign indicates the positive or negative effect of the SNP allele with higher frequency.
