# Supplementary figures and images for: Genetic dissection of the relationships between grain yield components by genome-wide association mapping in a collection of tetraploid wheats
Source: PLoS One. 2018 Jan 11;13(1):e0190162. doi: 10.1371/journal.pone.0190162 (PMC5764242; doi:10.1371/journal.pone.0190162)

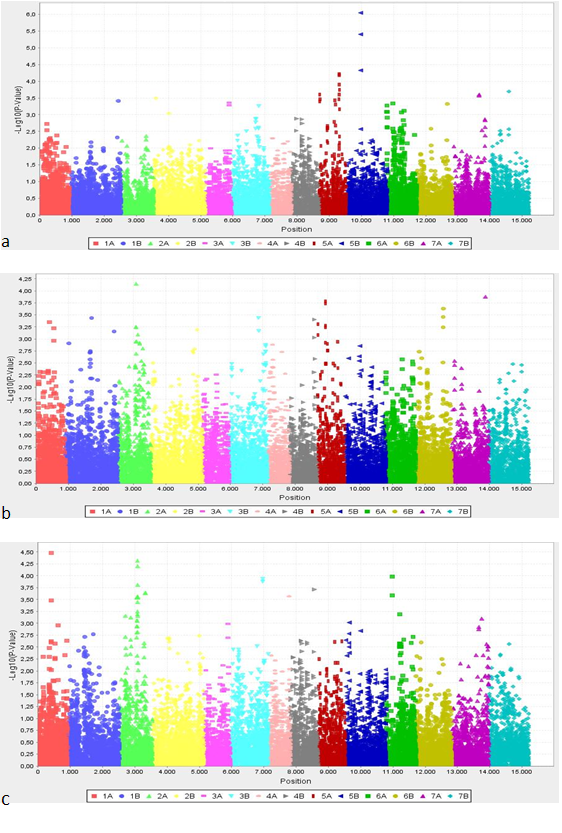

Supplement: S1 Fig — Manhattan plot from MLM+K model for the mean values across environments of grain yield per spike (a), thousand kernel weight (b) and number of kernels per spike (c). (TIF) [file pone.0190162.s004.tif]
